# Editorial: Insight and Intuition – Two Sides of the Same Coin?

**DOI:** 10.3389/fpsyg.2018.00689

**Published:** 2018-05-14

**Authors:** Michael Öllinger, Kirsten Volz, Eörs Szathmáry

**Affiliations:** ^1^Parmenides Foundation, Pullach im Isartal, Germany; ^2^Universität Tübingen, Tübingen, Germany

**Keywords:** insight, intuition, evolution, creativity, representational change

When we prepared this research topic, we had the strong feeling that there is a need to systematically investigate the relationship between insight and intuition. Although there have been approaches attempting to link these concepts (e.g., Bowers et al., 1990), we found several blind spots. Particularly, we missed a coherent model or at least well-defined and proper cognitive processes which unambiguously demarcate insight from intuition. We now have evidence from empirical and theoretical contributions which shed light on those blind spots.

All contributions agree that intuition and insight are based on distinguishable cognitive processes, but emphasized and detailed in part fairly different aspects. We are positive that our research topic will help to draw a clearer and more coherent picture, and inspire further research.

From a conceptual point of view, Zander et al. proposed that intuition is characterized by an experience-based and continuous process, whereas insight relies on a discontinuous process. An insight is realized by the problem solver all of a sudden, as if coming “out of the blue.” Given this assumption, they aimed at developing a paradigm in which insight and intuition could be investigated by the same tasks. They identified semantic coherence tasks as an ideal candidate for this challenge.

In the same vein Zhang et al. proposed the details of an experimental procedure which addresses the underlying processes of insight and intuition within a unified experimental paradigm. They also analyzed similarities and differences between these two processes. They focused on the different roles that tacit knowledge plays in both processes. Both the work of Zhang et al. and Zander et al. stressed the importance of a single paradigm allowing to investigate both processes to uncover the significant differences and similarities of insight and intuition.

Öllinger and von Müller proposed that coherence building and search might structure the problem-solving process determining a stage model. In their proposal, coherence building acts as base for intuition and insight. However, for the latter a change of the initial coherent representation is crucial. The change is driven by the realization after repeated failure that the initial problem representation cannot lead to the solution.

Pétervári et al. investigated the relationship between intuition and creativity. They reviewed the relevant literature and detected a strong link between the two concepts. The authors decomposed creativity into two separate processes—idea generation and idea evaluation, and linked intuition to the stage of idea generation. This investigation also highlighted an obvious link to dual process models detailing the interplay of exploitation and exploration, such as in AI and in various fields of biology (evolution and development) and might inform further research in these fields.

In sum, it seems that insight and intuition play different roles at different stages of the problem-solving process, differing by information integration, generation of hypotheses, search and eventually the change of representations. However, at this point empirical questions still remain: “What particular processes are underlying intuition and insight?” and “How could they be investigated?”. The following contributions elaborate on these questions.

Hedne et al. tested whether subjective feelings of intuitions are predictive for successful problem solving. During problem solving participants were asked to make metacognitive judgments. Insight trials showed a higher accuracy than non-insight trials. With their findings, the authors suggested that insight relies more on unconscious processes than non-insight problem solving. Insightful attempts are characterized by a deeper understanding of the solution. The authors speculate that at a metacognitive level problem solvers became aware that insightful solutions are more complete and better understood than non-insight solutions.

Complementarily, Gilhooly showed the importance of the unconscious work hypothesis for insightful solutions in an extensive literature review on incubation and creativity. The author contrasted the unconscious work hypothesis by alternative explanations and demonstrated convincingly that unconscious work becomes a driving factor and the main process for intuition and new insight during incubation—a phase during problem solving, in which participants do not make deliberate solution attempts.

The work of Dietrich and Haider proposes a new cognitive architecture which provides the key ingredients for answering the question: Why are our brains so creative? They detailed 10 foundational concepts, such as evolutionary algorithms which show the importance of recombining these concepts in a new and creative way. Prediction, scaffolding and competition of representations provide a dynamic which is sufficient for generating new candidate solutions, which were tested against a fitness function. Importantly, the authors also pointed out the open problems and further research questions, which have to be addressed in the future to complete the evolutionary picture of creativity.

Beyond mere phenomenology, Fedor et al. implemented a cognitive architecture, which is able to solve a difficult insight problem. The four-tree insight problem requires to overcome an ill-defined, over-constrained problem representation. This will lead to a larger search space which contains the solution. The framework is based on Darwinian Neurodynamics. The model evolved candidate solutions by replicating and evaluating neural representations in parallel. Emphatically, this parallel search must happen in the unconscious domain. The authors convincingly demonstrated that the model behaved comparable to human problem solvers.

Another key feature of insight is the Aha! experience. Little is known about the exact nature of this subjective experience. Clarifying the underlying processes might be crucial, since almost all neuroscientific studies rely on the pre-supposed relationship between Aha! and correct and insightful solutions.

Danek and Wiley scrutinized the question whether the Aha! experience is a reliable indicator for a correct and insightful solution. The authors proposed a multicomponent construct which decomposes the Aha! into distinct facets (suddenness, certainty, surprise, pleasure, etc.). Their study indicated that Aha! experiences were also found for incorrect solutions, and correct solution differed behaviorally (e.g., by faster solution times) from incorrect solutions.

Webb et al. were interested in the relationship between accuracy and Aha! ratings across problem types [insight, non-insight, compound remote associates (CRA)]. They found that classical insight problems elicited stronger Aha! experiences than hybrid types (like CRA), or non-insight problems. They demonstrated that an Aha! is elicited during an insightful problem-solving process and linked to accuracy.

Kizilirmak et al. shed light on the neural correlates which occur when insightful solutions were induced. Participants solved compound-remote-association tasks while lying in an fMRI scanner. The authors proposed that induced insight is the result of an interplay of detecting novel congruent schemata (medial prefrontal cortex) and the left hippocampus, which forms a novel meaning by the interrelatedness of familiar items. Additionally, positive memory effects of induced insight were found 24 h after the learning phase.

Finally, two contributions were interested in the notion: How do interventions affect intuition and insight? The first study trained thinking on contraries and the second addressed the interplay of intuitive processes and depression.

Branchini et al. addressed the question: How does training, which fosters thinking on contraries, influence the solution of insight problems. They applied the training either to small groups or individually. The main finding was that trained persons in small groups focused stronger on problem elements that were relevant for the solution. The study provides potential evidence that group processes might help to overcome self-imposed constraints.

Remmers and Michalak scrutinized the impact of depression on intuition. From their review of the relevant literature, they provided evidence of an impaired decision-making process in persons who suffer from depression. They stated that depression impedes coherent and holistic representations, resulting in unsatisfying states. Depression increases the likelihood for dysfunctional solutions that have negative behavioral consequences. They discussed potential treatments (e.g., metacognitive training) which might improve beneficial intuitive processes in depressive patients and reduce maladaptive intuitive processes.

In summary, these contributions to the research topic demonstrate convincingly how intuition and insight could be demarcated and modeled and provide a potentially productive paradigm for further research on this issue. There are still open questions: “Are insight and intuition different stages at the stream of problem solving (two sides of the same coin), or whether the two differ by the underlying processes such as discontinuous vs. continuous?”. An exciting period of further research lies ahead.

## Author contributions

MÖ wrote the editorial. KV and ES reviewed and edited the manuscript.

### Conflict of interest statement

The authors declare that the research was conducted in the absence of any commercial or financial relationships that could be construed as a potential conflict of interest.
